# Postoperative Submandibular Gland Swelling following Craniotomy under General Anesthesia

**DOI:** 10.1155/2015/949483

**Published:** 2015-11-30

**Authors:** Haruka Nakanishi, Tetsuya Tono, Shoichiro Ibusuki

**Affiliations:** ^1^Department of Otolaryngology-Head and Neck Surgery, Miyazaki University, 5200 Kihara, Kiyotake, Miyazaki 889-1692, Japan; ^2^Department of Anesthesiology, Miyazaki University, 5200 Kihara, Kiyotake, Miyazaki 889-1692, Japan

## Abstract

*Objective*. Reporting of a rare case of postoperative submandibular gland swelling following craniotomy.* Case Report*. A 33-year-old male underwent resection for a brain tumor under general anesthesia. The tumor was resected via a retrosigmoid suboccipital approach and the patient was placed in a lateral position with his face down and turned to the right. Slight swelling of the right submandibular gland was observed just after the surgery. Seven hours after surgery, edematous change around the submandibular gland worsened and he required emergent reintubation due to airway compromise. The cause of submandibular gland swelling seemed to be an obstruction of the salivary duct due to surgical positioning.* Conclusion*. Once submandibular swelling and edematous change around the submandibular gland occur, they can worsen and compromise the air way within several hours after operation. Adequate precaution must be taken for any predisposing skull-base surgery that requires strong cervical rotation and flexion.

## 1. Introduction

During retrosigmoid suboccipital approach the head may be positioned at the extreme limits of rotation and flexion to maximize anatomical exposure. In rare cases, such patients may develop swelling of the submandibular gland after operation.

Kim et al. [1] reported 5 cases of submandibular gland swelling following skull-base surgery, a prevalence of 0.84% based on all retrosigmoid and far-lateral craniotomy.

Singha and Chatterjee [2] reported a case of postoperative swelling of the submandibular gland following craniotomy. In their case, the submandibular gland swelling was contralateral to the craniotomy site which was the side most affected by the extreme positioning. The submandibular gland swelling was detected perioperatively; therefore they concluded mechanical obstruction as the inciting event for acute swelling of the submandibular gland.

In contrast, Hirai et al. [3] reported a case of postoperative submandibular gland swelling where the patient was in the supine position and no flexion of the neck during craniotomy. One possible reason that was considered was submandibular swelling as Wharton's duct obstruction due to an endotracheal tube or an increase in the volume of saliva due to anticholinesterase administration.

We report an unusual case in which acute swelling of the submandibular gland occurred after retrosigmoid suboccipital craniotomy for posterior fossa tumor under general anesthesia.

## 2. Case Report

A 33-year-old male patient (weight 56 kg; height 174 cm) presented complaining of faintness and shivering on the left side of the body that had been occurring for 2 months, associated with unsteadiness toward the left and diplopia that worsened when looking to the right.

**Figure 1:**
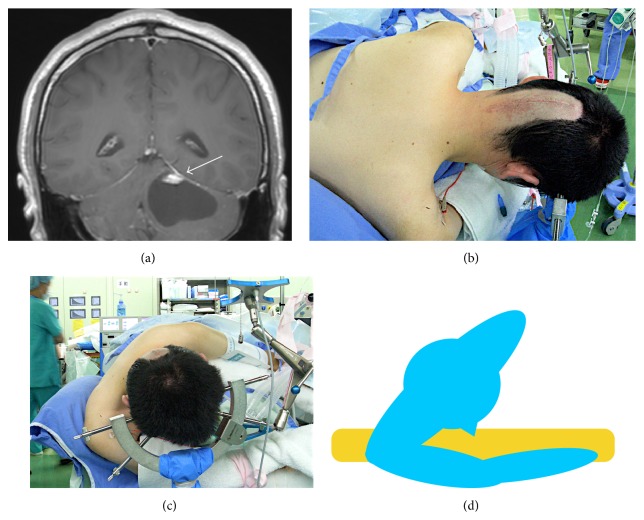
(a) Coronal image of preoperative MRI shows the tumor with cystic lesion on the left middle cerebellar peduncle (arrow). (b) Operative position was right lateral-semiprone position with chin down. (c) The right arm of the patient was placed in front of the neck to the left side and gently supported. (d) An illustrative diagram of the operative posture. Right submandibular lesion was caused by pressure being exerted on the face by this extreme positioning.

Magnetic resonance imaging showed a 12 × 13 × 9 mm extra-axial tumor lesion on the left middle cerebellar peduncle that accompanied a 24 × 35 × 21 mm cystic lesion under the tumor lesion (Figure 1).

The preoperative diagnosis was hemangioblastoma. The patient had no abnormal findings with regard to salivary glands on the preoperative computed tomography scan. A right retrosigmoid suboccipital craniotomy was planned to remove the tumor. Operative position was right lateral-semiprone position with chin down (Figure 1).

No anesthetic premedication was administrated. Anesthesia was induced with remifentanil with O_2_, air, and sevoflurane. Muscle relaxation was achieved with rocuronium bromide, and the patient was intubated uneventfully with a 8 mm internal diameter reinforced tube. To facilitate the surgical approach, the patient was placed in the extreme left lateral position and his sides were supported and pressure points secured.

The surgery proceeded uneventfully and all hemodynamic parameters as well as fluid input/output were maintained in the intraoperative period. At the end of the surgery, when the patient was found to be conscious and responding to commands, he was extubated in the operation theater. Total operation time was 4 hours and 23 minutes. Total anesthesia time was 6 hours and 8 minutes. Total infusion volume was 2,250 mL. Blood loss was 195 mL. Urine volume was 510 mL. Neurological assessment was satisfactory. However, a slight degree of right submandibular swelling was noticed after the extubation. An ultrasonic examination was performed by a dental surgeon who happened to be there at that time and pointed out the swelling of the right submandibular gland. As his vital signs were found to be within normal limits, he was permitted to leave the operation theater under close observation.

**Figure 2:**
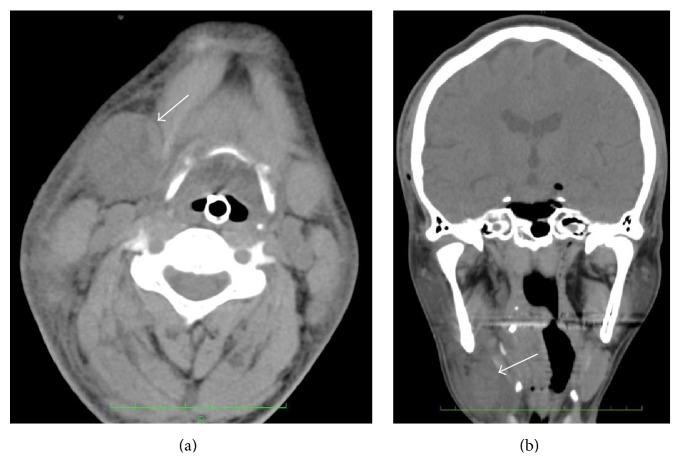
Axial (a) and coronal (b) images of computed tomography 1 hour after operation showing swelling of the right submandibular gland and the associated edematous tissue (arrow).

A computed tomography 1 hour after operation revealed swelling of the right submandibular gland and the associated edematous tissue. The submandibular gland was approximately 32 × 30 × 37 mm in size, consistency being firm, and the margins were well demarcated. No evidence of sialolithiasis was discovered (Figure 2).

**Figure 3:**
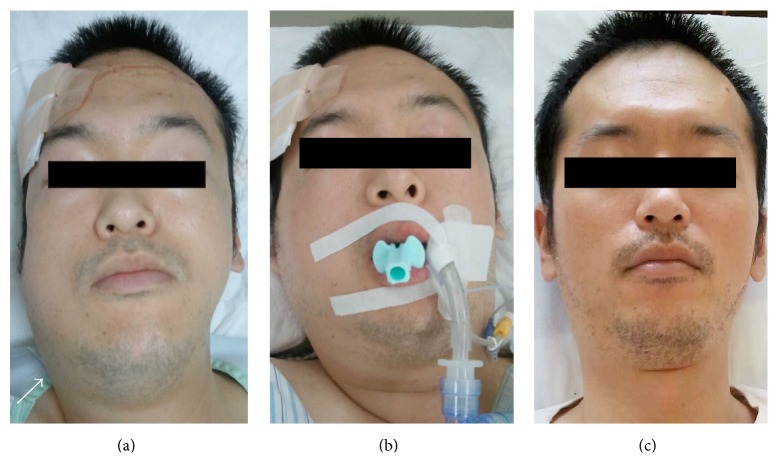
(a) Swelling of the right submandibular lesion (arrow) at 7 hours after operation. (b) Two days after operation, swelling of the submandibular lesion is still apparent. (c) The glandular swelling had almost disappeared by the fifth postoperative day.

**Figure 4:**
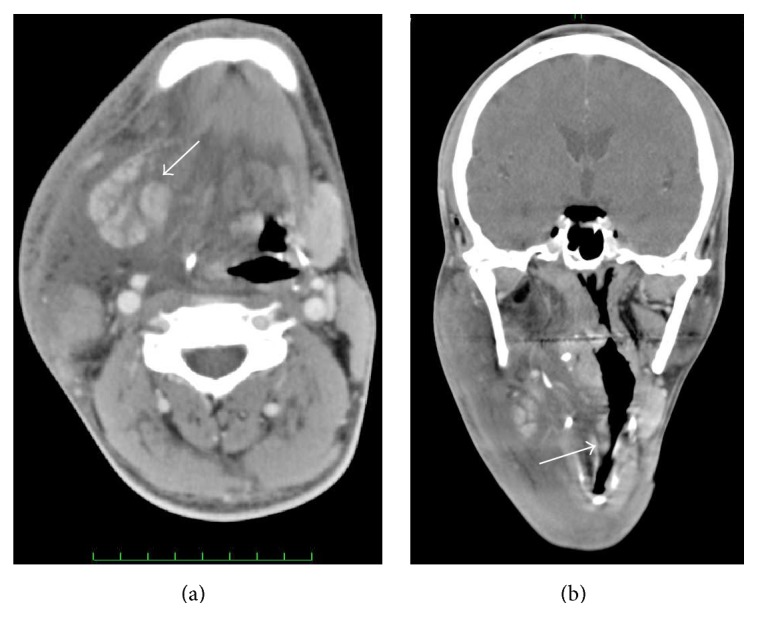
(a) Axial image of computed tomography 7 hours after operation. Edematous tissue around the right submandibular gland (arrow) was found to have increased, and it extended to the right parotid region. (b) Coronal image of computed tomography 7 hours after operation. The glandular swelling was found to be displacing the larynx (arrow).

7 hrs after operation, the swelling worsened and the patient started complaining of mild respiratory distress, although peripheral oxygen saturation (SpO_2_) was 100% and his vital signs were normal (Figure 3). A fiberscopy examination revealed apparent swelling of the right posterior part of the tongue. Computed tomography was performed again and revealed swelling of the right submandibular gland and also massive edematous change around the submandibular gland, extending to the right parotid region. Glandular swelling was found to be displacing the larynx (Figure 4). The patient was shifted to the intensive care unit and intubated soon after showing features of respiratory distress and in consideration of impending airway obstruction was put under mechanical ventilation for 5 days after the operation.

The swelling did not appreciably reduce during the first 48 hrs. Duration of intubation was based on clinical evaluation of soft tissue swelling, assessment of airway patency, and ultrasonographic examination.

The glandular swelling had almost disappeared on the fifth postoperative day, and he was extubated without any residual complications. After extubation, the patient did not require any further pulmonary care. The laboratory data showed the white blood cell count rose to 11,300/*μ*L and CRP also rose slightly to 0.86 mg/dL on the operation day and then decreased rapidly after operation. Serum amylase rose slightly to 186 IU/L on the day of the operation and decreased rapidly to within normal range while the submandibular gland was swollen. No other laboratory parameters that indicated comorbid disorder were associated with the submandibular swelling. SS-A and SS-B antibodies, antinuclear antibody, rheumatoid factor, LDH, IL-6, and IgG 4 were also within normal range. The result of bacterial cultivation from the floor of the oral cavity showed a small amount of* Pseudomonas aeruginosa*; however it seemed to have no relationship with the submandibular gland swelling because no purulent discharge or inflammation signs were observed around the sublingual caruncle. Third-generation cephalosporin antibiotics were given from the beginning of the operation to the fifth postoperative day. Betamethasone (4 mg/day) and glyceol (400 mg/day) were also given to reduce brain edema from the operation day to the fifth postoperative day. The pathological diagnosis of the brain tumor was hemangioblastoma.

## 3. Discussion

The association of skull-base surgery with acute swelling of the submandibular gland has rarely been reported, particularly in otolaryngological literature. The surgery lasted for 5 hrs, which is well within the average duration of skull-base surgical procedures at our institution. The submandibular swelling was detected perioperatively. This presentation supports mechanical obstruction as the inciting event for acute swelling of the submandibular gland in this case. This patient did not develop overt clinical signs of salivary gland infection. Considering the extreme rotation and flexion imparted during the retrosigmoid craniotomy procedures in the present patient, the mechanism of the development of the swelling was likely manifold. In our case, the swelling was found on the ipsilateral side of the craniotomy, which was the side most affected by the extreme positioning. It seems that compression of the tongue from the endotracheal tube and extreme head positioning during rotation and flexion can occlude Wharton's duct or cause ischemia or stasis of the submandibular gland and upper neck tissue around the submandibular gland. After the conclusion of the surgery, copious hydration aids the facilitation of salivary secretions and relieves the ischemia or stasis that may lead to acute swelling of the submandibular gland and massive edematous change due to reperfusion disorder. Ultrasonic examination revealed apparent dilatation of the intraglandular duct, yet serum amylase did not significantly rise during postoperative observation.

Reilly [4] reported parotid gland swelling that occurred after general anesthesia and named it “anesthesia mumps.” The prevalence was 0.2% of patients who underwent general anesthesia. The cause is generally considered to be associated with mechanical compression caused by airway devices and acceleration of salivary secretion during general anesthesia. In almost all cases of “anesthesia mumps,” serum amylase almost never increases and the swelling of the glandular lesion disappears in a few days. Regarding serum amylase invariability and the clinical course, our case resembled “anesthesia mumps.”

Some drugs used in general anesthesia increase the salivary secretion that can lead to swelling of the submandibular gland due to excessive salivary secretion if the salivary duct is obstructed [1, 5]. Succinylcholine, ketamine, and anticholinesterase are thought to have risk factors that increase the discharge of saliva. However, usage of these kinds of drugs has decreased because of the improvement of other anesthesia drugs and they were not used in our case.

Once submandibular swelling and edematous change around the submandibular gland occur, they can worsen and compromise the air way within several hours after operation and last several days even after immediate injection of steroids or antibiotics. Adequate precaution has to be taken for any predisposing skull-base surgery that requires strong cervical rotation and flexion.

## 4. Summary

Observation for perioperative cervical swelling should be performed, especially after operations that require strong cervical rotation and flexion.
